# Necrological

**Published:** 1881-04

**Authors:** 


### NECROLOGICAL.

"Death is the crown of life:
Were death denied, poor man would live in vain."

### 1839. RICHARD OSWALD COWLING. 1881.

On Saturday, April 2, 1881, at a few minutes past noon, DR. RICHARD OSWALD COWLING departed this life at his residence in this city.

For many weeks past he has been ailing. Rheumatism, an old enemy, had renewed its attack on his system, and with pain and difficulty he performed his daily duty. Intimate, personal and professional friends remonstrated against his continued self-neglect and plead that regular treatment might resist the assailant. Both the strong body dispised the subtle antagonist, and the brave heart could not consent that duty be left undone. Summoned to Cincinnati by the claims of official duty, he obeyed the call in spite of the earnest protest of his most trusted medical friend, and returned only to lie down on the bed from which he should rise up no more.

He did despise a fraud in whatever shape the counterfeit might come. He had no patience with the ignorance which arrayed itself in the robe of knowledge, and as little for the man who, calling himself the servant of humanity and of science, did yet measure his ministrations and his success by the columns of his ledger or the wealth and social position of his clients. He loved men and he loved his art. For these he would do all, dare all, suffer all; and in comparison with these money and its power were in his view as nothing. A great practice did not to him guarantee a scientific physician, nor a large bank-account a faithful follower of Hippocrates.

Perhaps no medical writer has more fully appreciated the wisdom of the Horatian maxim, "*Miscere utile cum dulci*," or has more successfully essayed its obedience. He wrote of things abstract, abstruse, and technical in a style so brilliant that even lay minds were interested in the perusal of his articles. He illustrated things difficult and dark by their analogy to things clear and easy of apprehension, so that dullness could understand and remember them. And in writing of subjects other than those purely scientific or professional, how beautifully did his medical knowledge come into play for ornament and illustration; and every where the man was apparent in the writing—the true man who hated shams and would speak the truth.

Longer, still longer, may we remember the noble vindication of the Christian faith against the "oppositions of science falsely so-called," which he spake as the Faculty's farewell to the graduates whom they were sending forth to labor for men. Yes, he believed that there is a good Father Whose we are and from Whom we came. Although his knife had dissected a thousand dead bodies, he had never found the hidden principle and potency of life. He believed that the science in whose study he rejoiced, and in whose triumphs he gloried, had not and cannot explain away our Creator—God, Who, His Son did come to tell us, is our Father. And therefore in words beautiful and strong *he declared* that a *scientific physician cannot be an atheist.*

Alas! that a dispensation of an Allwise God we have lost him! We mourn the loss of a man true, fearless, tender; of a physician thoughtful, diligent, studious; of a surgeon skillful, prudent, daring; of a writer cultivated, brilliant, profound; of a companion genial, instructive, buoyant; of a friend tried, true, and trusty.—*Louisville Medical News.*

